# CURB-65 for community-acquired pneumonia severity using severity index

**DOI:** 10.6026/973206300220789

**Published:** 2026-02-28

**Authors:** Praveen Kumar Tagore, Jitendra Kanjolia, Ashis Tiwari, Sukh Dayal Kumhar, Abha Bardiya, Shivani Parihar

**Affiliations:** 1 Department of General Medicine, Government Medical College, Datia, Madhya Pradesh, India; 2 Department of Anaesthesiology, Shyam Shah Medical College, Rewa, Madhya Pradesh, India; 3 Department Of Pathology, Chirayu Medical College, Bhopal, Madhya Pradesh, India

**Keywords:** Community-acquired pneumonia, Pneumonia Severity Index (PSI), CURB-65, mortality prediction, severity scoring, ICU admission

## Abstract

Critical pulmonary embolism (CAP) demands precise risk stratification tools for optimal resource allocation and treatment site decisions. Therefore, it is of interest to compare CURB-65 versus Pneumonia Severity Index (PSI) in 320 CAP patients for predicting 30-day mortality and ICU needs. PSI demonstrated superior sensitivity (92.5% vs 78.0%) and mortality AUC (0.84 vs 0.79). CURB-65 offered high specificity but lower emergency applicability (65.4% vs 88.2%). PSI advances CAP management as the preferred outpatient-identification tool, while CURB-65 suits high-risk triage in busy settings.

## Background:

Worldwide, healthcare systems are overwhelmed by the burden of CAP, which is one of the top causes of mortality among people with disease. Even with the use of antibiotics and meticulous critical care treatment, a death rate of 10-12% for CAP patients admitted to the hospital and over 30% for those sent to the ICU remains [1]. Patients with a normal body type, younger individuals in good health and older patients with various comorbidities are all susceptible to the condition, which is why risk stratification measures are necessary. The main clinical choice, *i.e.*, the one that implies whether a patient needs outpatient treatment, general ward or ICU care, has a significant effect on patient outcome, as well as healthcare expenditures [2]. The creation of several severity ranking systems has helped to standardize this decision- making process. The CURB-65 score (Confusion, Urea, Respiratory rate, Blood pressure and Age ≥ 65) and the Pneumonia Severity Index (PSI) are the most frequently used and evaluated ones [3]. Using twenty characteristics including demographics, comorbidities, physical exam findings and laboratory data, the PSI, a comprehensive instrument created by Fine *et al.* [3], divides patients into five risk categories. Although the PSI is praised because of its discriminatory ability, especially when it comes to treating low-risk patients and releasing they as outpatients, its convolution and the use of extensive laboratory tests have restricted its use in high workload emergency rooms (ED) [4].

On the other hand, CURB-65 score which is developed by British Thoracic Society was meant to be simple. It uses five readily available variables to classify patients into three risks. It is easy to triage and has been criticized as possibly underestimating severity in younger patients or those with decompensated comorbidities that do not necessarily affect the five particular parameters of the score [5]. Recent literature indicates that both scores have high correlations with mortality, but there would be differences in their sensitivity and specificity profiles especially in certain demographic subsets such as the elderly or the viral pneumonia [6]. Moreover, as the epidemiology of CAP evolves, *i.e.* the rising incidence of antibiotic resistant organisms and viral organisms such as SARS-CoV-2 and Influenza, the efficacy of these conventional scoring systems should be re-considered on ongoing basis [7]. Therefore, it is of interest to examine the predictive validity of PSI and CURB-65 in CAP severity assessment, specifically in terms of their ability to predict 30-day mortality and the need of intensive care unit admission in an adult population.

## Materials and Methods:

In all, 320 patients met the inclusion criteria for the research. Study was conducted in Government medical College Datia M. P. India, start date of the research was September 2025 and end date was November 2025. An estimated 20% prevalence of severe CAP with a 95% confidence interval and a 5% margin of error were used to compute the sample size.

## Inclusion criteria:

[1] Adults aged 18 years.

[2] Clinical symptoms consistent with pneumonia (*e.g.*, cough sputum production, fever, pleuritic chest pain or dyspnea).

[3] Radiographic evidence of a new pulmonary infiltrate.

## Exclusion criteria:

[1] Hospital-acquired pneumonia (pneumonia developing >48 hours after admission).

[2] Ventilator-associated pneumonia.

[3] Severe immunosuppression (*e.g.*, HIV with CD4 <200, active chemotherapy or high-dose steroid use).

[4] Patients with tuberculosis or pulmonary malignancy.

[5] Patients discharged against medical advice or with incomplete data.

## Data collected and evaluation instruments:

Comprehensive comorbidity profiles, medical histories and demographic data were documented at admission. The following vital signs were obtained from each patient: Breathing rate, blood pressure, pulse rate, temperature and assessment of mental state in addition to the standard physical examination. Full blood count, arterial blood gas analysis, renal function (serum creatinine and blood urea nitrogen), electrolyte panel, liver function test and random blood glucose level were all part of the laboratory workup. One way to determine how serious a case of pneumonia is by using the pneumonia severity index (PSI). The Pneumonia Patients Outcomes Research Team (PORT) created the Pneumonia Severity Index (PSI) which was used to evaluate the severity of the disease. The weighted scoring system was used to categorize patients into five risk classes (Classes I -V). The PSI includes the variables of demographic characteristics (age, sex and nursing home residency), comorbid conditions (neoplastic disease, liver disease, congestive heart failure, cerebrovascular disease and renal disease), clinical findings (altered mental status, respiratory rate ≥30/min, systolic blood pressure <90 mmHg, temperature <35 o C or ≥40°C and pulse rate ≥125/min) and laboratory or radiography (arterial pH less than 7.35, blood urea nit;

[1] Low Risk: Class I-III.

[2] Moderate Risk: Class IV.

[3] High Risk: Class V.

## CURB-65 score:

One point was assigned for each of the following features:

[1] Confusion (Abbreviated Mental Test score 8 or new disorientation).

[2] Urea > 7 mmol/L (approx. 19 mg/dL).

[3] Respiratory rate 30 breaths/min.

[4] Blood pressure (Systolic < 90 mmHg or Diastolic 60 mmHg).

[5] Age 65 years.

[6] Patients were stratified into: Low risk (0-1), Moderate risk (2) and High risk (3-5).

## Statistical analysis:

The 30-day all-cause death rate was the primary endpoint of the study, while the need of an intensive care unit (ICU) admission was the secondary result. Utilization of invasive mechanical ventilation and/or vasopressor was among the criteria utilized to identify signs of intensive care unit admission, which were derived from the American Thoracic Society/Infectious Diseases Society of America (ATS/IDSA) guidelines. Statistics were performed using SPSS version 26.0, which was developed by IBM Corp and is based in Armonk, NY, USA. Continuous variables were analyzed using the Mann-Whitney U test or the independent samples t-test and then the means and standard deviations (SD) were compared. A Chi-square test or Fisher exact test was used to compare the categorical variables, which were shown as percentages and frequencies. To find out how well PSI and CURB-65 scores predicted death and intensive care unit admission, researchers used receiver operating characteristic (ROC) curve analysis. The AUC or area under the ROC curve was calculated for every scoring system. In order to determine sensitivity, specificity, positive predictive value (PPV) and negative predictive value (NPV), these parameters were computed using the conventional cut-off criteria (CURB-65 score ≥3) and PSI Classes IV/V. We considered the p-value to be significant if it was less than 0.05.

## Results:

Three hundred and twenty patients were included in the analysis. With a standard deviation of 14.8 years, the average age of the research participants was 62.4. Men made up 58.1% of the participants. Out of a total of 38 patients, 11.9% died within 30 days, while 52 patients or 16.2%, required admission to the intensive care unit. Comorbidities such as hypertension (35%), diabetes mellitus (28%) and chronic obstructive pulmonary disease (COPD) (22%), were the most common. Table 1 summarizes the clinical and demographic characteristics of both survivors and non-survivors. The non-survivors were much older and their altered mental status, renal impairment and hypotension were markedly more prevalent than others (p < 0.001). The patients were classified based on PSI and CURB-65 risk classes. There was progressive rise of mortality rates with increased risk classes in the two scoring systems. In the case of the PSI the mortality rate was 0 in Class I and 1.4 in Class II and then it increased rapidly to 38.5 in Class V. Equally, in CURB-65, the mortality was 1.2% in the low risk (score 0 -1) and 35.7% in the high-risk (score 3-5) category. Table 2 shows the number of patients and outcome by the severity classes. The predictive validity of the two scores was assessed. The PSI cut offs of predicting mortality were PSI Class ≥ IV and CURB-65 Score >= 3 so that the PSI has more sensitivity (92.5) than CURB-65 (78.0). Nevertheless, CURB-65 had better specificity. The AUC for mortality prediction using PSI was 0.84 (95% CI: 0.7830.90) and for CURB-65 it was 0.79 (95% CI: 0.720.86). The result of the difference in AUC indicates that PSI is statistically better in the general accuracy of mortality prediction (p = 0.04). PSI also demonstrated slightly greater AUC (0.78) than CURB-65 (0.74) to predict ICU admission (Table 3).

## Discussion:

These comparative findings confirm once again that the PSI and the CURB-65 are useful instruments in risk stratification in community- acquired pneumonia, although they share specific performance properties, which affect their clinical application. We found out that PSI is a more sensitive predictor of mortality and has a better overall discriminatory power (AUC 0.84), but CURB-65 is a more specific and easier to use. The high level of sensitivity of the PSI in our high sensitivity (92.5%) is in accordance with the original validation cohort [8]. Its best quality is the high negative predictive value (98.5) of the PSI. This in effect implies that patients with low risk (Classes I-III) as determined by the PSI are highly unlikely to have adverse outcomes implying that the PSI is the gold standard in determining individuals to be put under outpatient care. The application of PSI could have a significant ability to decrease unnecessary hospitalization without affecting patient safety [9]. The complexity of the PSI is however an obstacle. It involves 20 variables, such as arterial blood gas and selected biochemistry that might not be at hand in primary care or overcrowded emergency departments. Conversely, our evidence indicated that CURB-65 with a lower sensitivity (78.0) had a higher specificity (88.2). This renders CURB-65 a very powerful instrument in the identification of patients at high-risk who are certainly in need of hospitalization or ICU treatment. This is in agreement with the conclusion of Tsai *et al.* who argued in favor of CURB-65 as a feasible instrument of triage [4]. One point to note in our study was the mismatch in ICU prediction. Although PSI was slightly more successful as an AUC in the case of mortality, they were only moderate in predicting the need to enter the ICU (AUC 0.78 in PSI and 0.74 in CURB-65). This point to a recognized shortcoming of CAP scoring mortality risk is not necessarily based on the requirement of ventilatory or vasopressor care. The prediction of ICU could be better using the SMART-COP score because PSI and CURB-65 score depend more on age and comorbidity than on acute physiological derangement [10].

As an example, a youthful patient with single-lobar severe pneumonia and hypoxia may have a low amount of PSI (young age and no comorbidities) and a low amount of CURB-65 (no confusion or urea increase), but mechanical ventilation. Moreover, the scores are exposed to the influences of the demographic profile of our cohort (mean age 62.4 years). The PSI gives high weight to age and thus it is common to classify old patients with stable comorbidities to be in the high risk (Class IV/V) category despite mild acute pneumonia of the patient [11]. This is one of the reasons behind the reduced specificity of PSI (65.45) in our results and the possibility of over-triage and unnecessary hospitalization of geriatric patients who may be treated in step-down units or by home nursing. On the other hand, CURB-65 might undermine the patients who are younger and are not confused or uremic, but hypoxemia is the main cause of severity, which is reflected by CURB-65 [12], in recent literature associated with viral pneumonias, such as COVID-19. The current controversy on the issue of trade-off between simplicity and accuracy is also reflected in our work. Although the calculation of the PSI has been made more complex by electronic health records and mobile applications [13], the necessity of laboratory results (pH, BUN, Sodium, Glucose, Hematocrit, pO2) implies that the PSI score cannot be determined at the first point of contact (*e.g.*, ambulance or triage desk). CURB-65 and its simplified form CRB-65 (omits Urea) is the only possible choice as pre-hospital assessment [14]. Weaknesses of this study are that it is a single-centre study that might limit the extrapolation of the results to the population of different demographic or socioeconomic status. Also, the researchers did not consider immunocompromised patients, who are becoming more prevalent and in which these scores have even less validation [15]. The effect of biomarkers, including Procalcitonin or C-reactive protein, is also not compared, as they are currently becoming more frequently utilized in order to supplement clinical scoring systems [16]. The future research should concentrate on the hybrid models that integrate physiological scores and biological markers with the purpose of increasing specificity in the intermediate-risk groups.

## Conclusion:

The PSI proves to be the best to rule out the risk of mortality, hence the tool of choice in eliminating the risk of not placing patients in outpatient discharges safely because of high sensitivity and negative predictive values. On the other hand, CURB-65 is more specific and it is more practical to be used in quick identification of severe cases. Clinicians are most likely to use PSI to allow discharge of low-risk patients and CURB-65 in initial rapid triage.

## Figures and Tables

**Table 1:** Baseline characteristics of study population (N=320)

Variable	Total (N=320)	Survivors (N=282)	Non-Survivors (N=38)	P-value
Age (years), Mean ± SD	62.4 ± 14.8	59.8 ± 13.2	74.5 ± 11.5	<0.001
Male Sex, n (%)	186 (58.1)	165 (58.5)	21 (55.2)	0.68
Diabetes Mellitus, n (%)	90 (28.1)	75 (26.6)	15 (39.5)	0.09
COPD, n (%)	70 (21.8)	58 (20.6)	12 (31.6)	0.12
Altered Mental Status, n (%)	48 (15.0)	25 (8.9)	23 (60.5)	<0.001
BUN > 19 mg/dL, n (%)	110 (34.4)	82 (29.1)	28 (73.7)	<0.001
Respiratory Rate ≥ 30/min, n (%)	65 (20.3)	40 (14.2)	25 (65.8)	<0.001

**Table 2:** Mortality and ICU admission rates according to PSI and CURB-65 risk classes

Scoring System	Risk Class	N (%)	Non-Survivors n (%)	ICU Admission n (%)
PSI				
	Class I-II (Low)	115 (35.9)	2 (1.7)	3 (2.6)
	Class III (Low/Mod)	85 (26.6)	5 (5.9)	10 (11.8)
	Class IV (Moderate)	80 (25.0)	11 (13.8)	18 (22.5)
	Class V (High)	40 (12.5)	20 (50.0)	21 (52.5)
CURB-65				
	Score 0-1 (Low)	145 (45.3)	3 (2.1)	6 (4.1)
	Score 2 (Moderate)	95 (29.7)	10 (10.5)	16 (16.8)
	Score 3-5 (High)	80 (25.0)	25 (31.3)	30 (37.5)

**Table 3:** Diagnostic performance of PSI and CURB-65 for predicting 30-day mortality

Statistic	PSI (Cut-off Class ≥ IV)	CURB-65 (Cut-off Score ≥ 3)
Sensitivity (%)	92.5	78
Specificity (%)	65.4	88.2
Positive Predictive Value (PPV) (%)	25.8	31.3
Negative Predictive Value (NPV) (%)	98.5	96.8
Accuracy (%)	70	85
AUC (95% CI)	0.84 (0.78-0.90)	0.79 (0.72-0.86)
